# Toll-Like Receptor 9 Activation Rescues Impaired Antibody Response in Needle-free Intradermal DNA Vaccination

**DOI:** 10.1038/srep33564

**Published:** 2016-09-23

**Authors:** Prabhu S. Arunachalam, Ria Mishra, Krithika Badarinath, Deepak Selvam, Sravan K. Payeli, Richard R. Stout, Udaykumar Ranga

**Affiliations:** 1 HIV – AIDS Laboratory, Molecular Biology and Genetics Unit, Jawaharlal Nehru Center for Advanced Scientific Research, Bengaluru, India; 2 Institute for Stem Cell Biology and Regenerative Medicine, Bengaluru, India; 3 Bioject Medical Technologies Inc., Oregon, USA

## Abstract

The delivery of plasmid DNA to the skin can target distinct subsets of dermal dendritic cells to confer a superior immune response. The needle-free immunization technology offers a reliable, safe and efficient means to administer intradermal (ID) injections. We report here that the ID injection of DNA vectors using an NF device (NF-ID) elicits a superior cell-mediated immune response, at much lesser DNA dosage, comparable in magnitude to the traditional intramuscular immunization. However, the humoral response is significantly impaired, possibly at the stage of B cell isotype switching. We found that the NF-ID administration deposits the DNA primarily on the epidermis resulting in a rapid loss of the DNA as well as the synthesized antigen due to the faster regeneration rate of the skin layers. Therefore, despite the immune-rich nature of the skin, the NF-ID immunization of DNA vectors may be limited by the impaired humoral response. Additional booster injections are required to augment the antibody response. As an alternative and a viable solution, we rescued the IgG response by coadministration of a Toll-like receptor 9 agonist, among other adjuvants examined. Our work has important implication for the optimization of the emerging needle-free technology for ID immunization.

The route of delivery constitutes an important parameter defining the outcome of an immunization procedure. The skin containing a complex network of diverse subsets of immune cells interacting with the epithelial cells^1^,^2^ forms a preferred site for vaccination^3^. Of all the different antigen-presenting cells (APC) located in the skin^4^, the dermal dendritic cells (dDC) are of special interest due to the heterogeneity of the dDC subsets and the specialized antigen presenting functions of each subset^5^,^6^,^7^. The delivery of vaccine candidates to the skin, targeting specified DC subsets could elicit immune responses of superior quality in comparison to the traditional subcutaneous (SC) or intramuscular (IM) route of immunization.

The intradermal (ID) immunization using a needle and syringe proved quite efficient in inducing protective immune responses against tuberculosis^8^; however, the search for an alternate route of administration has been considered necessary due to various concerns^9^. The needle-based ID immunization is not a preferred strategy of vaccination for technical reasons including the difficulty in delivering large volumes^3^ and excessive inflammatory reactions at the site of ID injection due to the presence of adjuvants in the formulation^10^. The recent technical advances in the delivery of antigens to the skin using the needle-free (NF) devices^11^,^12^ revived the interest in the ID immunization. The ID immunization using an NF device such as Biojector 2000 (B2000) is reliable, reproducible and does not require substantial technical expertise. In addition to simplifying the procedure of immunization, the NF devices improve the safety profile of the vaccination^13^ and enhance the immunogenicity of vaccines^14^,^15^.

The DNA vaccines have been traditionally administered to the muscle via the intramuscular (IM) immunization. The IM administration of the plasmid DNA could induce an efficient immune response in small experimental animals, but the efficacy is limited in larger animals and human beings. The potency and immunogenicity of the DNA vaccines have been enhanced by delivering the encoded antigens to DC^16^ and by coadministering chemokines that induce DC maturation^17^. Unlike the muscle, the skin may offer a more suitable compartment for the administration of DNA vaccines due to the rich presence of the dDC subsets thus leading to an efficient immune response in the larger animals. Indeed, a large number of previous studies attempted to take advantage of the rich immune profile of the skin by delivering the plasmid DNA to the skin^18^,^19^,^20^,^21^,^22^,^23^. Although these attempts achieved a marginal success, the real potential of the skin immunization has not been appreciated given the difficulties of reproducibly administering the ID injection using the needle-syringe assembly and the technical limitation associated with the gene gun-mediated immunization. In comparison with the conventional IM immunization or the needle-dependent ID immunization, the NF-ID administration of the plasmid DNA has several technical merits. First, the needle-free devices can disperse the plasmid DNA to a relatively larger surface area of the skin making the encoded antigen accessible to a larger number of skin DC. Second, the use of adjuvants such as the Toll-like receptor (TLR) agonists in the formulation could help in tailoring a desired immune response by targeting a specific subset of the dDC as different dDC subsets vary significantly in the expression profile of the TLRs^24^. Lastly, the ID immunization mandates the use of a relatively low antigen/adjuvant dosage that constitutes an important concern for commercial vaccine development. Additionally, the NF-ID delivery offers a simple and reliable means to explore the innate mechanisms, especially of the dDC subsets, that orchestrate the adaptive immune responses, thus helping in rational vaccine design.

In this backdrop, given the technical merits of the NF devices, and biological significance, we set out to optimize the delivery of plasmid DNA encoding specific antigens to the skin using the NF-ID immunization. We show that inspite of inducing optimal cell-mediated immune (CMI) responses, the NF-ID immunization failed to elicit an efficient humoral immune response. We show further that the rapid recycling of the skin layers primarily underlies the suboptimal humoral immune response following the NF-ID administration. The antigen-specific serum IgG, but not the IgM, response was abrogated indicating a defective process of isotype switching. Importantly, we rescued the antibody response by coadministering the CpG ODN 2395, a class C TLR9 ligand (TLR9L), providing a viable solution to the problem.

## Results

### The IM and NF-ID immunizations elicit cell-mediated immune responses of comparable magnitude

**Figure 1:**
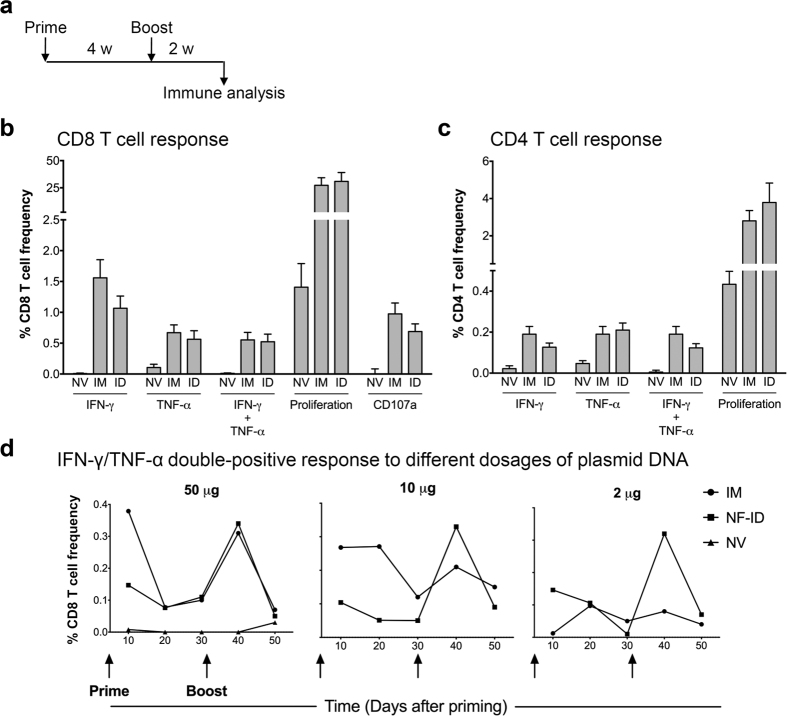
Comparable magnitude of cell-mediated immune responses elicited by the IM and NF-ID immunizations. (**a**) A line diagram showing the immunization schematic. Groups of female C57BL/6 mice were immunized with 50 μg null vector (NV) or pCMV-Gag vector. The null vector was administered through the IM route and the pCMV-Gag vector through the IM or NF-ID route. Two weeks after a booster injection, the splenocytes were harvested and subjected to the ICS, CD107a degranulation and CFSE dilution assays. The percentage frequencies of the CD8 (**b**) and CD4 (**c**) T cells manifesting the phenotypes depicted are plotted (mean ± SEM). The data are obtained from three independent experiments with four mice each per group. Unpaired *t* test was used for statistical comparison. The differences between the IM and NF-ID (labeled as ID) groups are not statistically significant. (**d**) Female C57BL/6 mice (n = 3) were immunized with 2, 10 or 50 μg of the pCMV-Gag expression vector at the time points as indicated by the arrows. The PBL collected every 10 days from all the mice in a group were pooled and analyzed for the cytokine expression using the ICS technique. The frequencies of the IFN-γ/TNF-α double positive CD8 T cells identified at each time points are plotted. The data are representative of two independent experiments. The two-way ANOVA was used for the statistical comparison and the differences between groups are not statistically significant.

To evaluate the efficacy of the NF-ID immunization to elicit CMI responses, we immunized batches of C57BL/6 and BALB/c mice with DNA expression vectors encoding HIV-1 Gag antigen through the conventional IM or the NF-ID route using the one-prime-one-boost immunization schedule as depicted (Fig. 1a). The Gag-specific T cell, CD8 and CD4, responses were measured from the splenocytes of the immunized mice using multi-parametric flow cytometry. The antigen-induced pro-inflammatory cytokine secretion, the cell-killing activity and the proliferative response of the activated T cells were evaluated. The CD107a degranulation was used as a surrogate marker for the cell-killing activity^25^. The flow cytometry profiles used for the analysis of IFN-γ and TNF-α response are presented as a representative of the measured CMI responses (Fig. S1a). Regardless of the route, the Gag immunization elicited a superior CD8 T cell response, several fold higher in magnitude, in comparison to the null vector control (Fig. 1b). The frequency of the antigen-responsive CD8 T cells secreting a single cytokine, IFN-γ or TNF-α, or both the cytokines is comparable between the IM and NF-ID groups with the differences being statistically insignificant. The IFN-γ/TNF-α double-positive CD8 T cell frequencies elicited by the IM and NF-ID immunizations were 0.55 ± 0.12% and 0.52 ± 0.12%, respectively. Likewise, the CD8 T cell proliferative response and CD107a degranulation were also comparable between the two routes of immunization. The induced immune responses in the CD4 T cell compartment were also comparable between the two routes of immunization although the overall magnitude of the CD4 immune response was relatively low as compared to that of the CD8 compartment. The cytokine response, single or dual cytokine induction, and the proliferative response of the CD4 T cells induced by the NF-ID and IM immunizations did not differ significantly from each other (Fig. 1c). The IFN-γ/TNF-α double positive CD4 T cell frequencies elicited by the IM and NF-ID immunizations were 0.19 ± 0.04 and 0.12 ± 0.02%, respectively. In addition to the splenocytes, we also measured the CD8 T cell response in the draining lymph nodes and found that the IFN-γ/TNF-α double-positive response, measured as a representative of the CMI responses, to be comparable between the IM and NF-ID routes, although lesser in magnitude as compared to that of the splenic T cells (Fig. S1b). Furthermore, we also compared the effect of the routes of immunization in BALB/c mice that are Th-2 predisposed. The induction of the pro-inflammatory cytokines and CD107a degranulation response were comparable between the IM and NF-ID routes of immunization also in the BALB/c mice (Fig. S1c).

The ID immunization can confer dose sparing effect on several distinct vaccines^26^. We, therefore, examined if the NF-ID immunization could induce efficient immune response at a reduced DNA dosage as compared to the IM injection. We immunized C57BL/6 mice with 2, 10 or 50 μg of the vector encoding HIV-1 Gag and measured the frequency of the IFN-γ and TNF-α double-positive CD8 T cells in the peripheral blood of the mice. The blood collected through the retro-orbital puncture was pooled within a group, 200–250 μl of the blood obtained from each animal. The red blood cells (RBC) were lysed using the ammonium chloride lysis buffer, and the peripheral blood leukocytes (PBL) obtained following extensive washing were used in the cytokine analysis. The strategy of using the PBL, rather than the splenocytes/lymph node cells, in the immune analysis described above offers the great advantage of collecting repeated samples from the same animals in a longitudinal experimental follow up without sacrificing the animals. The dual cytokine response of IFN-γ and TNF-α secretion was monitored up to day 50 following priming. Ten days after the priming, at a dosage of 50 μg, the cytokine response was higher in IM, as compared to that of the NF-ID immunization (Fig. 1d). The CD8 T cell response, however, reduced considerably in 20 days after the priming by either route. In spite of the significant differences observed at the priming, following the administration of the booster immunization at day 30, the profile of the immune response at day 40 was similar and indistinguishable between the routes of immunization. Importantly, the effect of the reduced DNA dosage at 10 and 2 μg was quite profound on the IM route. As the DNA concentration reduced, the secondary immune response too diminished substantially with almost no cytokine response evident at the 2 μg dose in the IM injection. In contrast, the secondary immune response sustained in the case of NF-ID route with a comparable magnitude of cytokine induction manifested at all the three dosages of the DNA including 2 μg in spite of the low level primary immune response. Thus, the NF-ID route appears to elicit a superior magnitude of immune response at a relatively low concentration of the plasmid DNA.

### The NF-ID immunization results in a suboptimal humoral immune response to the DNA-encoded antigens

**Figure 2:**
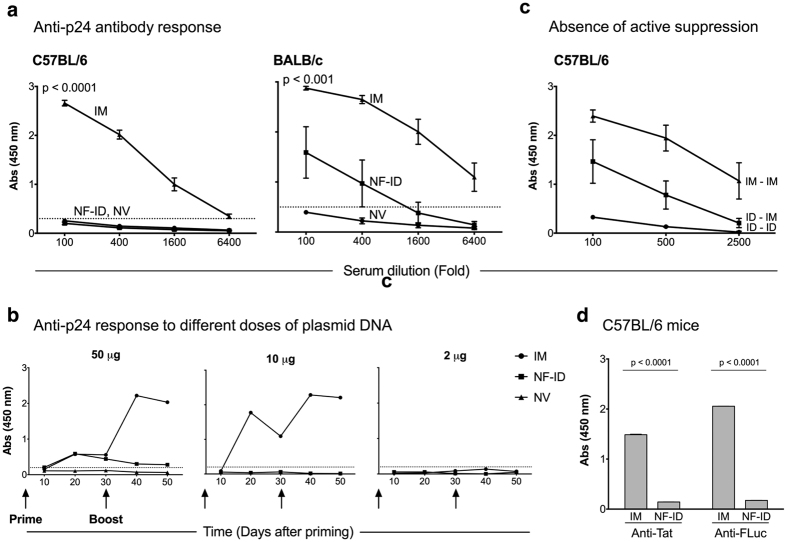
Suboptimal humoral immune response to DNA-encoded antigens following the NF-ID immunization. (**a**) Groups of mice, C57BL/6 (n = 12, left panel) or BALB/c (n = 4–6, right panel), were primed and four weeks later boosted. The blood samples were collected after 11 days following the booster immunization (See Fig. 1a for the immunization schematic). The titers of the Gag-specific antibodies were determined using an indirect ELISA. The absorbance values (mean ± SEM) of the serially diluted serum samples are plotted. The dotted lines represent the cut-off value of the assay (mean + 2SD of the null vector group). The data are representative of several independent experiments. The statistical difference between the NF-ID and IM routes was compared at the 100-fold dilution using the unpaired *t* test. (**b**) The blood of the C57BL/6 mice immunized as in Fig. 1c collected at different time points, the plasma samples pooled within a group, assessed by ELISA at a 100-fold dilution, and the absorbance values are plotted. The dotted line represents the cut-off value defined as two times the mean absorbance value of the control group immunized with the null vector. The two-way ANOVA was used for the statistical comparison. The differences between the IM and NF-ID immunization of 50 and 10 μg DNA are significant (p < 0.05). (**c**) The antibody response in the C57BL/6 mice primed by the NF-ID route and boosted by the IM route. The absorbance values of the sera diluted serially (100 to 2500-fold) are plotted (mean ± SEM, n = 4). The ID in the labels refers to NF-ID. (**d**) The sera of C57BL/6 mice (n = 4) immunized with 50 μg pcDNA3.1-Tat or pCMV-FLuc, as in Fig. 1a, were pooled, diluted 100-fold and evaluated for antigen-specific antibody response by ELISA. The absorbance values (mean ± SD) were plotted and the data are representative of two independent experiments.

An efficient immunization is expected to induce both the arms of the immune system, the cell-mediated and the humoral responses. Having confirmed the induction of high quality CMI response (Fig. 1), we asked if the NF-ID immunization could elicit an optimal humoral immune response. Antibodies alone or in combination with the CMI can constitute the protective component of the immune response against many infections^27^. Using ELISA, we determined the titers of Gag-specific antibody response in the sera of mice immunized via the IM or NF-ID route. While the sera of mice immunized via the IM route contained high titer antigen-specific antibodies, as expected, the presence of anti-Gag antibodies could not be identified in the sera of the NF-ID mice (Fig. 2a). The sera of C57BL/6 mice immunized with 50 μg of the plasmid DNA through the IM route contained anti-Gag antibodies up to a serum dilution of 6,400. In contrast, even at a dilution of 100, the NF-ID sera failed to show any measurable antibodies (Fig. 2a, left panel). Even at a lower dilution of the serum samples, we could not detect an antibody response in the NF-ID group and these sera were indistinguishable from those of the null vector immunization (Fig. S2). Although the DNA vaccines are usually not efficient in eliciting a high quantity humoral immune response, the contrast between the IM and NF-ID routes is quite striking and significant (p < 0.0001) suggesting a severe impairment in the induction of the antibody response by the NF-ID route. Unlike C57BL/6, the BALB/c mice tend to manifest a Th2 type biased immune profile that should favor a stronger humoral immune response. We, therefore, immunized BALB/c mice and examined the anti-Gag antibody response in the sera. In the BALB/c mice, a measurable antibody response was generated in response to the NF-ID immunization (Fig. 2a, right panel). This response, however, was significantly inferior to that of the IM immunization (p < 0.001). The data of the BALB/c immunization are consistent with those of C57BL/6 and are suggestive of a severe compromise, if not abrogation, of the antibody response. At a lower DNA dose (10 μg of plasmid DNA), the difference between the IM and NF-ID immunizations was consistent in C57BL/6 mice ruling out the possibility of a potential immune tolerance or exhaustion underlying the unresponsive nature of the NF-ID route (Fig. 2b)^23^.

Next, to understand if an active mechanism of B cell suppression impeded the antibody response in the NF-ID arm, we used a mixed immunization strategy. A group of mice was primed through the NF-ID route and boosted via the IM route (ID-IM), in addition to the immunization of the regular IM-IM and ID-ID groups. The IM-IM and the ID-ID immunizations induced high and low titers of anti-Gag antibody responses, respectively, as before (Fig. 2c). The immunization of NF-ID priming followed by the IM immunization produced a humoral immune response of an intermediate magnitude alluding to the absence of an active mechanism of suppression initiated at priming by the NF-ID immunization. Importantly, the defective humoral immune response of the NF-ID route was not restricted to HIV-1 Gag but was manifested against two other experimental antigens. We used DNA expression vectors encoding HIV-1 Tat, a poor and mostly intracellular antigen, or firefly luciferase (FLuc), a common experimental antigen. The NF-ID immunization as compared to the IM, failed to induce a measurable antibody response against either of the antigens in C57BL/6 mice (Fig. 2d). Collectively, the data suggested an impaired humoral, but not cell-mediated, immune response to DNA-encoded antigens delivered via the NF-ID route.

### The ID immunization elicits IgM antibodies but does not induce isotype switching

**Figure 3:**
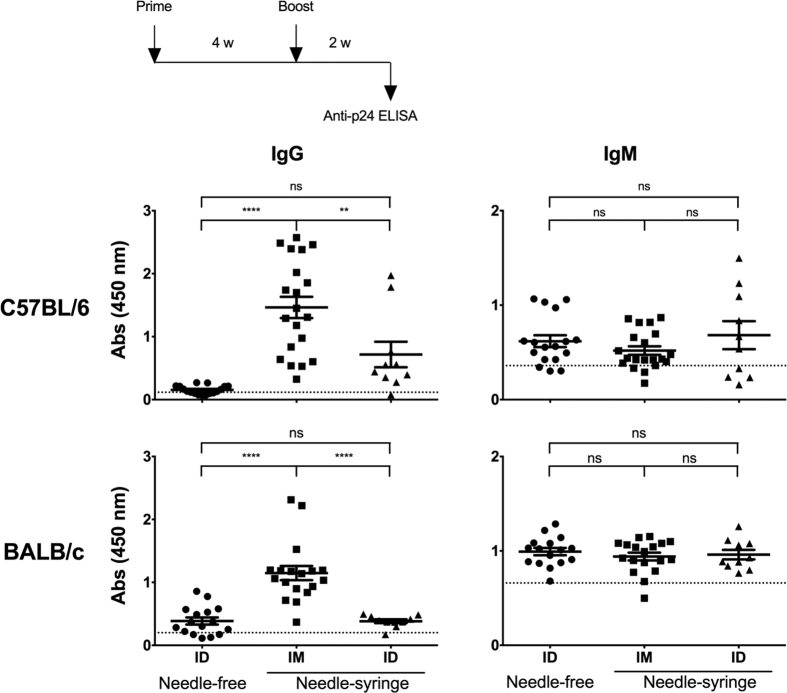
Suboptimal IgG, but not the IgM, antibody response following the NF-ID immunization. Groups of mice, C57BL/6 or BALB/c, were immunized with the pCMV-Gag DNA vector through the NF-ID, or needle-syringe IM or ID route. Each symbol represents an individual animal. The sera obtained 11 days following the booster immunization were diluted 100 and 500-fold for the measurement of IgG in C57BL/6 and BALB/c mice respectively. The IgM from both the strains of mice was measured in serum diluted 100-fold. The assay employed secondary antibodies conjugated to HRP specific to IgM or IgG isotypes. The mean absorbance value of the group and the SEM are shown. The dotted line represents the cut-off value defined as two times the mean absorbance value of the control group of four animals immunized with the null vector. The data of the NF-ID and IM groups were obtained from three independent experiments containing 4–6 mice per group. The data of the needle-syringe ID group were obtained from two independent experiments containing 4 or 6 animals per group. The one-way ANOVA was used for statistical comparison (****p < 0.0001 and **p < 0.001).

Importantly, the immune assays used in the previous experiments were designed to detect the presence of only the antigen-specific IgG antibodies. As the secondary antibody used in the ELISA was raised against mouse IgG, these immune assays precluded the detection of antigen-specific IgM antibody response. We, therefore, used an IgM-responsive ELISA to examine if the defect of the NF-ID immunization was at the level of induction of the antigen-specific IgM antibody response. We simultaneously measured the antigen-specific IgG antibody response in the mouse antisera. Furthermore, in addition to the IM and NF-ID modes of immunization, we added a third mode of immunization. The ID administration using the needle and syringe (NS-ID) was added to rule out the possibility that the non-invasive NF-ID immunization was unable to induce the damage/danger-associated molecular pattern (DAMP) mediated signaling that may be essential for eliciting an inflammatory response^28^. Three groups each of C57BL/6 or BALB/c mice were immunized, the serum samples were collected 11 days after the booster immunization, and the Gag-specific IgM and IgG antibody responses were measured in the sera (Fig. 3). In C57BL/6 mice, the anti-Gag IgG antibody response via the IM route was significantly superior (mean absorbance 1.46 ± 0.17) to the NF-ID (0.15 ± 0.02). Importantly, the profile of the immune response of the NS-ID immunization was low (0.72 ± 0.2, p < 0.05) and statistically comparable to that of the NF-ID suggesting that regardless of the use of the needle in the immunization procedure, when the DNA is delivered to the skin, the antibody response is compromised. Thus, the biological nature of the target compartment appears to be the primary factor influencing the outcome of the immunization (Fig. 3, top panels). Comparable results were obtained in BALB/c mice (Fig. 3, bottom panels). When the IgM antibody response was measured, the mean antibody titers between the IM and ID routes were comparable and no defective IgM response was seen in either of the ID immunizations. Especially in BALB/c mice, the mean absorbance values of either of the ID immunization (0.99 ± 0.04 and 0.96 ± 0.05 in NF-ID and NS-ID immunizations, respectively) was comparable to that of the IM group (0.94 ± 0.04). The antigen-specificity of the IgM response was verified by coating a non-specific antigen in the ELISA (Fig. S3). Collectively, the data are suggestive that the administration of plasmid DNA into the skin of mice is capable of eliciting high quality CMI response and antigen-specific IgM antibodies at levels comparable to that of the IM immunization. The ID immunization, however, fails to drive the switching of the antibodies from IgM to IgG. Thus, the introduction of plasmid DNA in the skin of mice, either by the NF-ID or NS-ID mode, causes a severe impairment in the antibody response as compared to the IM route in two different strains of mice.

### The antigen expression is suboptimal following the NF-ID immunization

Given that only the B cell, but not the T cell, immune responses were attenuated in the NF-ID immunization, we conjectured that the suboptimal B cell immune response could be the result of insufficient amount of antigen available in the skin. The B cell response typically requires larger quantities of antigen^29^. To this end, we generated reporter DNA vectors expressing two different luciferase proteins. While Gaussia luciferase (GLuc) is secreted from the cells therefore is systemically circulated, the firefly luciferase (FLuc) is intracellular and can be measured at the site of injection. The expression of the luciferase *in vivo* can offer a sensitive and, importantly, a quantitative assay to examine the kinetics of the protein expression following the administration of the DNA-expression vectors.

**Figure 4:**
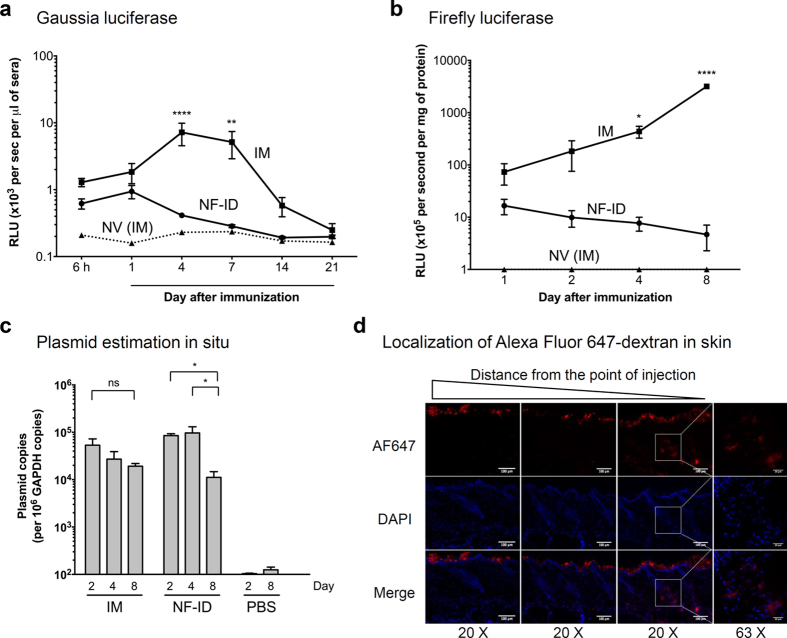
Short-lived antigen expression and rapid DNA elimination following NF-ID immunization. BALB/c mice were injected with 50 μg of the GLuc or FLuc expression vectors or the null vector through the IM or NF-ID route. (**a**) The GLuc-administered mice were bled at different time points as shown on the X-axis. The serum samples were diluted 10-fold, the luciferase enzyme activity was measured and the data are presented as mean absorbance ± SEM, n = 6. The data are representative of three independent experiments. (**b**) In the FLuc group, the sites of injection were collected at different points as shown, and the tissues were pulverized and lysed to obtain the whole tissue homogenate. A total protein of 50 μg of the tissue extract was used for the luciferase assay. The luciferase activity was converted into the enzyme activity per mg of the protein and plotted as the mean ± SEM, n = 4. The data are representative of two independent experiments. The two-way ANOVA was used for the statistical evaluation. (**c**) The quantitative real-time PCR analysis of the total DNA extracted from the muscle or skin tissue following the immunization of the pCMV-Gag DNA vector. A 106 bp region of the CMV promoter was amplified and normalized using the GAPDH control amplified in parallel. The data are presented as the mean ± SEM plasmid copy number per million GAPDH copies. The data are representative of two independent experiments. The two-way ANOVA was used for the statistical comparison (*p < 0.05, **p < 0.001 and ****p < 0.0001). (**d**) BALB/c mice administered with 20 μg of Alexa Fluor 647-labelled dextran using NF-ID were sacrificed 8 h post injection. The 10 μm thick cryosections of the skin tissue were stained for DAPI and the representative images captured at three different areas were presented.

Groups of mice were injected with GLuc or FLuc expression vectors or an empty vector, via the IM or NF-ID route, and the expression profile of the luciferase proteins was determined at different time points from the mouse serum samples (GLuc) or in the tissue homogenate (FLuc). The presence of GLuc in the mouse serum samples was detected as early as 6 h in both the groups of DNA administration. The enzyme activity was low and comparable between the two groups at 6 h and day 1. At day 1, the mean absorbance values of GLuc were 1840 ± 600, 940 ± 210 and 158 ± 7 RLU/s in the IM, NF-ID and NV groups of mice, respectively (Fig. 4a). The enzyme activity in the IM group increased and peaked at day 4 or 7 and reduced progressively thereafter and touched the baseline by day 21. In contrast, the enzyme activity of GLuc in the NF-ID mice did not increase after day 1 and only reduced thereafter and by day 7 the levels were comparable to those of the empty vector control group. On day 4 when the GLuc enzyme activity peaked in the IM group, the enzyme activities between the IM and NF-ID groups were significantly different (7190 ± 266 and 410 ± 20 RLU/s, respectively, and p < 0.0001). To assess the protein expression of FLuc at the site of injection, we estimated the FLuc activity from the muscle or skin tissue homogenates up to day 8. The expression profile of FLuc in the tissue extracts at the site of injection was similar to that of the GLuc (Fig. 4b). While at day 1, the enzyme activities between the IM and NF-ID routes were modestly different (73.37 ± 32.26 × 10^5^ and 16.57 ± 5.42 × 10^5^, respectively), the enzyme activity thereafter followed a digressing profile progressively increasing in the IM group and reducing in the NF-ID group up to day 8. At day 8, the mean enzyme activity of the IM and NF-ID was found to be 3.18 ± 0.3 × 10^8^ and 4.67 ± 2.39 × 10^5^ RLU/s, respectively. GLuc apparently disappeared from blood at a much faster rate than that of the FLuc from the muscle tissue, potentially due to neutralizing antibodies in the blood^30^. Collectively, the expression profiles of two different luciferases are consistent with each other and with the previously published data^31^, and confirmed a rapid reduction of either of the enzymes when the DNA expression vectors were administered into the skin, but not into the muscle.

### Potential cause(s) underlying the loss of skin-expressed proteins

The data of FLuc expression are especially informative (Fig. 4b). The enzyme activity of FLuc in the skin was not as high as that of the muscle, even at day 1 when the differences were not statistically significant. The suboptimal enzyme activity in the skin could be a net outcome of defects at multiple levels including the instability of the plasmid DNA and the expressed protein in the skin, and the relatively modest transcriptional activity of the CMV promoter in the skin cells. The epidermis of the mouse skin comprises of three layers of epithelial keratinocytes spanning a thickness of less than 20 μM. The top layer of the epidermis is replaced every 7–8 days^32^. Therefore, a large quantity of DNA introduced into the skin may be subjected to physical elimination within 8 days unlike the DNA injected into the muscle tissue where the DNA could survive for a longer duration. Using PCR to amplify a 106 bp region of the CMV promoter on the plasmid, we quantitated the number of plasmid DNA molecules present in the skin and the muscle at the site of administration at different time points, up to eight days (Fig. 4c). On day 2, the plasmid copy number was comparable between the IM and NF-ID immunizations, 5.33 ± 1.92 × 10^4^ and 8.52 ± 0.79 × 10^4^ copies, respectively. The plasmid copy number reduced marginally in the muscle on days 4 and 8. On day 8, in IM immunization, the plasmid number reduced to 1.92 ± 0.27 × 10^4^ copies that translated to a 65% loss of the plasmid DNA and this loss was not found to be statistically significant. In contrast, the loss of plasmid DNA in the NF-ID immunization was insignificant between days 2 and 4, but a precipitating loss was noted on day 8 resulting in the presence of only 1.12 ± 0.35 × 10^4^ copies. This corresponds to a loss as high as 90% of DNA originally injected into the skin that was found to be statistically significant (p < 0.05). Even though a significant loss was found only at day 8, the antigen expression was limiting as early as day 4. The inhibited antigen expression may also have been due to the type-I interferon mediated signaling as reported previously^33^.

A loss of more than 90% of the DNA compounded with a suboptimal antigen expression suggested that the DNA injected through the NF-ID technique was delivered to the epidermis primarily. To evaluate this possibility, we administered Alexa Fluor 647-labelled dextran, an anionic fluorescent molecule, by NF-ID and examined the localization of the fluorescence signal using confocal microscopy of the cryosections of the skin. We find that the bulk of fluorescence was localized on the top of epidermis of the skin 8 h following administration (Fig. 4d). Only a minor proportion of the fluorescence could be found in the dermis. The data demonstrate the inability of the jet injection technique to effectively surpass the epidermal layers of the mouse skin. The data of the luciferase expression, DNA quantitation and immunohistochemistry of the injected antigen collectively are suggestive of a rapid loss of the DNA and the expressed antigen in the skin compartment as a consequence of the skin shedding possibly contributing to the observed suboptimal humoral immune responses in the NF-ID immunization.

### Additional booster immunizations or coadministration of a TLR9 agonist augments the humoral immune response in NF-ID immunization

**Figure 5:**
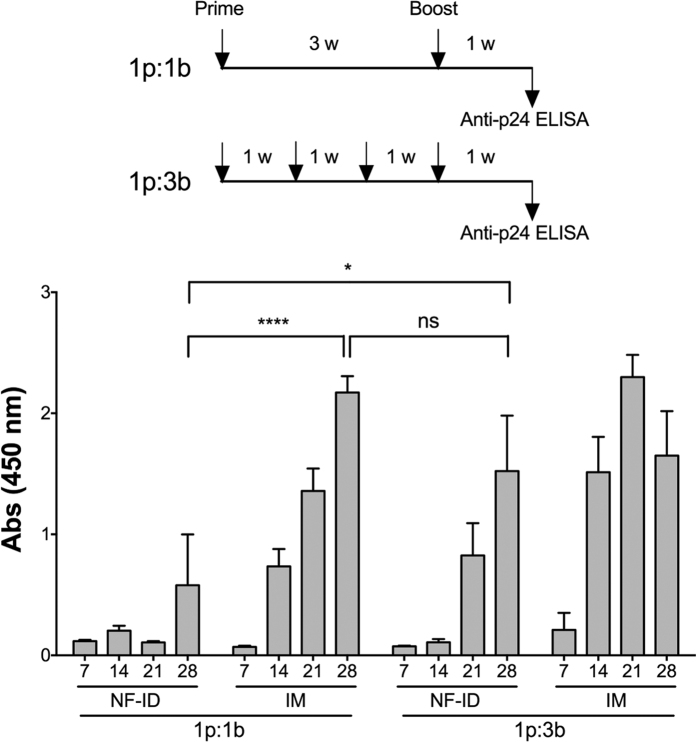
The repeated booster NF-ID immunization augments the IgG antibody response. A schematic representation of the prime-boost regimen used for the immunization of groups of male BALB/c mice (four animals per group) depicted at the top. The immunization schedules consisted of a single booster immunization or three booster doses administered within the same time frame. The animals were immunized with 50 μg of the pCMV-Gag expression vector at each immunization. The bar chart represents the anti-Gag IgG response measured from the serum of the immunized mice at the time points indicated in the X-axis. The means of the raw absorbance values obtained from the serum diluted 100 fold were plotted with SEM, n = 4. Two-way ANOVA was used for statistical evaluation (*p < 0.05 and ****p < 0.0001).

In the backdrop of suboptimal antigen expression following the NF-ID immunization, we reasoned that if the rapid loss of the antigen from the skin compartment indeed leads to the suboptimal humoral immune response, the administration of additional booster immunizations within a fixed time frame should restore the immune response. To test this hypothesis, we immunized two groups of mice, for each of the IM and NF-ID routes, one with the standard one-prime-one-booster immunization regimen (1P:1B) and the other with two additional booster immunizations (1P:3B) and compared the humoral immune response between the two groups as depicted (Fig. 5). The ELISA titers at day 28 were considered for the statistical comparison. The anti-Gag antibody response in the NF-ID immunization enhanced significantly when two additional booster immunizations were delivered. The absorbance value of 0.58 ± 0.42 of the 1P:1B immunization on day 28 increased to 1.52 ± 0.45 in the 1P:3B. Importantly, the enhanced antibody response of the 1P:3B regimen was statistically comparable to the 1P:1B IM immunization (2.17 ± 0.13). Furthermore, an incremental response was manifested in the NF-ID 1P:3B immunization at days 21 and 28. A positive correlation between the number of immunizations and the augmented immune response was evident when a smaller quantity of the plasmid DNA, 15 instead of 50 μg, was delivered at each administration (Fig. S4).

**Figure 6:**
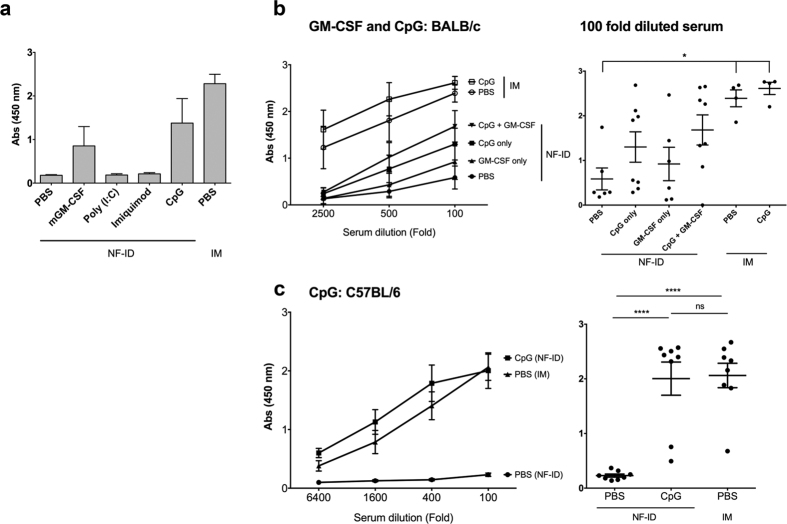
The adjuvant coadministration restores the IgG antibody response following the NF-ID immunization. (**a**) BALB/c mice were immunized with 50 μg pCMV-Gag DNA in PBS and one of the several adjuvants as shown, 1 μg recombinant mGM-CSF, 30 μg poly (I:C), 25 μg Imiquimod, or 5 μg CpG ODN 2395. The vector immunization via IM served as a positive control for comparison. Two weeks after the booster immunization, the antibody response was measured in sera diluted 100-fold. The absorbance mean ± SEM values are plotted, n = 4. (**b**) BALB/c (n = 4–8) or (**c**) C57BL/6 (n = 8) mice were immunized with 50 μg pCMV-Gag DNA vector alone or in combination with the CpG ODN 2395 and/or GM-CSF. The anti-p24 IgG response was determined in ELISA. The means ± SEM of the absorbance values of the groups or the absorbance values obtained from individual serum samples (diluted 100-fold) are plotted in the left and right panels, respectively. The statistical evaluation was carried out using One-way ANOVA (*p < 0.05 and ****p < 0.0001).

Adjuvants, especially the toll-like receptor (TLR) agonists, have the potential to enhance or modulate immune responses. We tested if the coadministration of agonists specific to different TLRs, along with the DNA vectors, could enhance the quality of the immune response to NF-ID when an immunization regimen consisting of only a single booster is administered. All the immunization protocols described above have used only the naked DNA administration in the absence of any immune modulator. To this end, we immunized groups of mice with HIV-1 Gag-expression DNA vector in the absence or the presence of mGM-CSF (a DC differentiation factor), poly (I:C) (a TLR3 agonist), imiquimod (a TLR7 agonist), or class C CpG ODN 2395 (a TLR9 agonist). The IM immunization of the plasmid DNA was included as the positive control. In BALB/c mice, following the NF-ID immunization, the coadministration of mGM-CSF or CpG ODN 2395, but not poly (I:C) or imiquimod, could augment antibody response to p24 as compared to the no adjuvant control (Fig. 6a). The mean absorbance value of the DNA immunization in PBS 0.18 ± 0.05 enhanced to 0.85 ± 0.44 and 1.34 ± 0.56 with the coadministration of mGM-CSF and the CpG ODN, respectively. To see if there would be a synergistic response between the two immune-modulators, we immunized fresh batches of BALB/c mice with HIV-1 Gag expression vector in the presence of one or both of the immune-modulators (Fig. 6b). There was a remarkable restoration of the antibody response in the presence of CpG ODN which did not enhance further to a significant extent by the addition of mGM-CSF. The combined effect of the presence of both the immune-modulators of NF-ID still did not match the antibody response of the IM immunization. Interestingly, the coadministration of CpG ODN did not enhance the antibody response further in the IM immunization, unlike in the case of the NF-ID. In NF-ID immunization of BALB/c mice, despite an explicit increase in the antibody response, the difference in the titers observed on the adjuvant communization did not reach statistical significance given the high magnitude variability among the animals. On the other hand, a remarkable restoration of the antibody response was observed in the C57BL/6 mice, which was statistically significant. The mean absorbance value of the NF-ID immunization in PBS 0.23 ± 0.02 increased to 2.0 ± 0.3 when the CpG ODN was coadministered (Fig. 6c). Importantly, the immune response of the NF-ID in C57BL/6 mice was consistent, reproducible and comparable to that of the IM immunization (2.06 ± 0.22, p < 0.05). Furthermore, the quality of the T cell response was consistent and did not enhance further due to the immune-modulator (Fig. S5). In the blood, the frequency of p24-specific CD8 T cells double positive for IFN-γ and TNF-α remained comparable between the IM and NF-ID groups coadministered with or without CpG ODN, 0.17, 0.16, 0.24 and 0.16% in the IM, IM + CpG, NF-ID and NF-ID + CpG groups respectively.

## Discussion

The primary objective of the present study was to examine the immunization potential of the NF-ID route as compared to that of the IM route to deliver DNA-expression vectors. The superiority of the ID route of immunization over the IM route has been well established in many elegantly designed studies previously^23^,^34^. The ID immunization is effective for several different vaccines, and currently, the BCG, rabies, and an influenza vaccine are administered intradermally^34^. The skin is a preferred compartment of immunization due to the presence of diverse subsets of DC specializing in antigen presentation. However, the majority of the previous studies delivered the DNA vectors using the needle-syringe assembly or via the gene gun device. Given the technical difficulties involved in administering the ID injections reproducibly and the lack of the safety profile, the NS-ID immunization is not a preferred strategy for the delivery of the DNA to the skin. Furthermore, needle-based immunization is not conducive to the mass vaccination programs. The NF devices, therefore, have been progressively gaining prominence especially for delivering the DNA vaccines to the skin.

Two different NF strategies are available for the delivery of the DNA vectors to the skin, the particle-mediated bombardment, more popularly known as the gene gun, and the particle-free administration^11^. The gene gun mediated administration of the plasmid DNA coated on gold particles has been a popular strategy for the DNA vaccine delivery^35^,^36^; however, this immunization technique is known to predispose the antibody response towards a Th2-type immune profile^37^,^38^. The particle-free ID immunization is shown to elicit a balanced Th1/Th2 immune response superior to that of the IM immunization^39^. Additionally, the soluble immunogens employed in the NF-ID technique allow the coadministration of a large number of adjuvants. Furthermore, the application of an NF device such as B2000, the only device available commercially for the ID immunization of mouse, requires limited technical skills and training.

Inspite of the technical merits, the immunization efficiency of the NF device has not been subjected to rigorous experimental evaluation. Of the modest number of studies that evaluated NF-ID immunization of plasmid DNA vectors^39^,^40^,^41^,^42^, we found few studies that compared the NF-ID immunization to a conventional route such as IM in parallel. We therefore, attempted to fill this gap by comparing the NF-ID immunization with the conventional IM as well as the NS-ID immunizations using a variety of DNA-encoded antigens. Our results confirmed the findings of the previous publications that the NF-ID is highly efficient in inducing the CMI response to the encoded antigens. The magnitude of the CMI response elicited by the NF-ID is comparable to or even surpassed that of the IM immunization, especially at a lower DNA dosage, in which the NF-ID route elicited a stronger immune response (Fig. 1d). Despite eliciting an efficient CMI response, the NF-ID immunization was found to be inefficient in inducing the humoral arm of the immune response to at least three different proteins (Fig. 2). This result is in contrast to Meseda *et al.*^39^, who demonstrated an efficient antibody response to the HSV-2 glycoprotein D, but similar to Brave *et al.*^40^, who showed an anti-HIV-1 p24 titer of about 500 following three successive NF-ID injections using the B2000 device. The observed discordance could probably be explained by the nature of protein; whether or not the antigen is membrane-bound. Brave *et al.* demonstrated a relatively higher titre of antibodies to the HIV-1 Env protein which is membrane-bound in comparison to the p24 protein that is cytoplasmic^40^. Thus, the data from the present study as well as from a few previous reports appear to confirm our findings. The absence of a comparison between the NF-ID and the IM route in the previous studies does not, however, permit a meaningful interpretation of the results.

The absence of efficient humoral immune response in the NS-ID immunization (Fig. 3) appears to suggest a suboptimal nature of the ID route per se in mouse. Caution, however, must be applied in the backdrop of previous studies demonstrating the superiority of ID over the IM immunization^18^,^31^,^36^,^43^,^44^,^45^. In mouse, the ID injection was reported at three different sites using a needle-syringe assembly: the ear pinnae, the dorsal flank of the skin (NS-ID) and the base of the tail. The ID immunization delivered to the ear pinnae proved to be the most efficient route^31^,^46^ followed by that of the tail immunization^18^,^43^ both being superior to that of the IM immunization in eliciting antibody response. In contrast, the NS-ID immunization of the flank of the skin doesn’t seem to be as efficient as the two other NS-ID modes or even IM in eliciting an antibody immune response^31^,^36^,^44^ consistent with our own data. Collectively, the NS-ID immunization of the flank of the mouse appears to be inefficient in eliciting antibody response as compared to IM although only one study made a direct comparison of these two modes of immunization^36^ to the best of our knowledge.

Having confirmed that the NF-ID route is efficient in inducing the cell-mediated, but not the humoral, immune responses in mice, we explored for a possible explanation. Several lines of evidence are indicative that the persistence of antigen expression in skin could be a limiting factor determining the quantity of humoral immune response induced. The most important clue emerged from the observation that the IgG, but not IgM, titers were reduced in the NF-ID immunization as compared to IM suggesting a deficiency at the level of CD4 T cell help in mediating the isotype switch (Fig. 3). Whether a continued presence of the antigen expression is necessary for the induction of an optimal CD4 T cell immune response is controversial. Despite the conflicting results, several studies demonstrated that the duration of antigen expression plays a deterministic role in shaping the magnitude of the CD4 T cell immune response that may in turn determine the magnitude of the antibody response^47^. In contrast to our data, Forg *et al.* demonstrated a higher antibody response to β-galactosidase following the ID immunization in ear pinnae^31^. The moderately higher antibody response in this study could be attributed to the relatively higher antigen concentration produced throughout the duration of antigen expression following the ear pinnae immunization in comparison to the NF-ID immunization^46^. The persistence and magnitude of firefly or Gaussia luciferase in our experiments were comparatively shorter following the NF-ID immunization (Fig. 4). Thus, transient expression of the antigens in skin layers may have been sufficient for the induction of the antigen-specific IgM response, but insufficient to induce the class switching to IgG as a consequence of suboptimal CD4 T cell help.

The epidermal layers of the skin are replaced at a high rate due to sloughing off of the skin^48^. In the mouse, the recycling rate of epidermis is seven days resulting in a progressive regeneration of the outermost layers^32^. The rapid loss of epidermal layers could underlie a concomitant elimination of the administered plasmid DNA as well as the expressed antigens, thus, compromising the quality of humoral immune response. We evaluated this hypothesis by administering a larger number of booster immunizations within the same duration of the immunization schedule. The antigen-specific IgG antibody titers improved significantly as more booster immunizations were administered thereby validating the hypothesis (Fig. 5). The antigen concentration plays an important role in the B cell receptor (BCR) engagement and the strength of the signaling transmitted. Thus, the suboptimal concentration of antigen may be sufficient to induce the differentiation of B cells into plasma cells and the extra-follicular isotype switching, but insufficient to cause the engagement of the B cells and the follicular T helper cells, an interaction critical to induce the isotype switching and the germinal center reactions^49^. We acknowledge that our data are limiting in not evaluating this possibility. Nevertheless, we demonstrate convincingly the outcome of this process, which represents the nature of the antigen-specific antibodies IgG versus IgM. We are presently investigating the mechanism of the impaired B cell isotype switching following NF-ID immunization.

Apart from the rapid loss of plasmid DNA and the antigen from the skin, additional innate suppressive mechanisms influencing the overall outcome must be considered. Unlike the APC of muscle tissues, the dermal DC represent the most potent innate immune cells to sense the presence of foreign plasmid DNA in the skin and respond by activating a variety of integrated stress responses^50^. One such mechanism could be the induction of a strong type-I interferon response resulting in inhibition of the antigen synthesis, similar to what has been reported in a recent study using the recombinant adenoviral vectors^33^. The promoter silencing is yet an additional mechanism causing low antigen production^51^,^52^. The CMV promoter, however, is strongly functional in the epidermal keratinocytes and the dermal cells^53^. We nevertheless compared the mouse EF1-α promoter with the CMV promoter and found a comparable result interms of the lack of an efficient antibody response in the NF-ID immunization (Data not shown).

Of note, the gene gun immunization targets the epidermis of the skin primarily, similar to the NF-ID immunization with B2000. Yet, these seemingly comparable immunization strategies appear to yield a contrasting antibody response. While the gene gun elicits an antibody response typically stronger than that of the IM route^35^, the NF-ID is found to induce a low antibody response^40^,^42^. The gene gun immunization delivers DNA to the nuclei of epidermal cells including the Langerhans cells (LC) thus actively promoting gene expression in almost all the transfected cells. The transfected LC migrate to the draining lymph nodes and induce a B cell response^48^. In contrast, the NF-ID injection by B2000 deposits the plasmid on the epidermal layers of the skin (Fig. 4d). The uptake of the deposited DNA requires macropinocytosis or phagocytosis by the LC and other cells. Our attempts to detect antigen expressing cells in the skin or antigen-presenting cells in the lymph node have not been successful probably due to these technical challenges. Thus, the limiting DNA uptake and compromised antigen expression in the NF-ID, but not gene gun, collectively underlie an impaired antibody response although over 90% antigen is lost within a week following both the techniques^48^,^54^. Our data emphasize on the immediate need to optimize the NF-ID jet administration strategy to enable the delivery of the DNA to the dermal cells surpassing the barrier of the epidermis.

Importantly, in addition to identifying the cause underlying the suboptimal humoral immune response following the NF-ID immunization, we demonstrated a strategy to restore the antibody response, the coadministration of a TLR9-agonist within the standard one-prime-one-boost regimen. The TLR-agonists and cytokines such as GM-CSF and IL12 are gaining importance as adjuvants because of their ability to enhance or modulate the immune responses^55^,^56^,^57^. We tested three different TLR-agonists and GM-CSF to augment the antibody response. Brave *et al.* reported an enhancement of the antibody response after three successive injections of GM-CSF^40^. In our hands, however, the GM-CSF demonstrated only a moderate efficacy although we used the same form of the cytokine as Brave *et al.* Glycosylation at the N-term of the GM-CSF protein is known to make the cytokine more potent for the *in vivo* applications although both non-glycosylated and glycosylated GM-CSF are similar in the property of cell differentiation *in vitro*^58^. Of all the TLR agonists evaluated, the TLR9-agonist CpG ODN 2395, efficient in stimulating B cell responses^59^, restored the antibody response with the highest efficiency in the NF-ID immunization (Fig. 6). Of note, the augmentation of antibody response by the ODN 2395 is significantly superior in C57BL/6 compared to BALB/c mice alluding to the inherent host differences between the mouse strains. The C57BL/6 mice, as compared to BALB/c, are also more prone to the ODN 2395-mediated toxicity, especially when injected through the NF-ID route, mandating the use of smaller dosages for the IM immunizations.

In summary, we demonstrated for the first time, a compromised humoral immune response to DNA vaccines in the NF-ID immunization. We showed that the faster regeneration rate of the skin layers, leading to a rapid loss of the administered plasmid DNA with a concomitant loss of the encoded antigen, as the underlying factor causing the antibody deficit in NF-ID immunization. Whether the suboptimal antibody response can prove a potential challenge in larger animals and the human beings needs additional evaluation. The epidermis is thicker in larger animals offering further difficulty in targeting the dermis through jet injection. Two previous studies, one in the human beings and the other in the macaques, employed the NF-ID route to administer DNA vectors^60^,^61^. In agreement with our results, these studies failed to detect an antibody response to two different protein antigens despite the induction of efficient T cell responses. Further, in the macaques, the NF-ID route proved to be a superior mode of priming for the subsequent MVA-mediated antibody response. In these studies, however, even the IM administration failed to elicit an antibody response indicating the need for further optimization of the DNA vaccines in large animals. Importantly, we demonstrated that the coadministration of a potential adjuvant could restore the antibody response following a single booster immunization. The potential of various other immune-modulators must be evaluated to induce an optimal and balanced immune response through the NF-ID route. Thus, our data have significant implications for the optimization of the emerging NF immunization technology.

## Materials and Methods

### Plasmid DNA vectors and reagents

The nucleotide sequences coding for the HIV-1 Gag, firefly and Gaussia luciferase proteins were cloned into the pNGVL3 vector backbone (University of Michigan, http://www.violinet.org/dnavaxdb/plasmid_detail.php?c_dv_plasmid_id=100)^62^. The HIV-1 *tat* gene was cloned in the pcDNA3.1 vector backbone (Cat #V920-20, Invitrogen, USA). The sequences of the HIV-1 *gag* and *tat* were of the consensus subtype C *gag* and *tat*, generated from 150 sequences representative of different countries downloaded from the HIV sequence database^63^,^64^. The *tat* gene was further engineered with the engraftment of the PanDR epitope (AKFVAAWTLKAAA) into the cysteine rich domain and the insertion of a synthetic intron obtained from pIRES puro (Cat #631605, Clontech Laboratories, USA) between exons 1 and 2^63^,^65^. Furthermore, the consensus subtype C gene sequences were codon-optimized for mammalian expression using the online codon optimization tool JCat (http://www.jcat.de). The codon-optimized sequences were chemically synthesized by the commercial vendor ShineGene Molecular Biotech Inc, China. The firefly and Gaussia luciferase genes were amplified using PCR from the commercial vectors pGL3 (E1751, Promega USA) and pGLuc-Basic2 (N8082, New England Biolabs, USA), respectively. The firefly *luc* gene was amplified using the primer pair N2392 5′-CTCT*GAATTC*GCCGCCGCCATGGAAGACGCCAAAAACATAAAG-3′ and N2393 5′-GGGCCC*TCTAGA*TTACACGGCGATCTTTCCGC-3′ using the amplification conditions 94^30”^-60^30”^-72^90”^ for 15 cycles. The Gaussia *luc* gene was amplified using the primer pair N2394 5′-CTCT*GAATTC*GCCGCCGCCATGGGAGTCAAAGTTCTGTTTGC-3′ and N2395 5′-GGGCCC*TCTAGA*TTAGTCACCACCGGCCCC-3′ with the PCR conditions 94^30”^-60^30”^-72^20”^ for 15 cycles. All the amplified expression cassettes were cloned between *EcoRI* and *XbaI* (the restriction sites were highlighted in the primer sequences in italics) in respective vectors under the control of the CMV promoter. The protein expression of Gag and Tat was confirmed in Western-blot from the lysates of transiently transfected HEK293T cells. The expression of firefly and Gaussia luciferase proteins was confirmed in a luciferase assay from the cell lysate or culture supernatant, respectively, of the transfected HEK293T cells. The plasmid DNA vectors used in the immunization were purified using the Qiagen endotoxin free Giga kit (Cat #12391, Qiagen, Germany). The endotoxin concentration in the purified plasmid stocks was found to be less than 0.05 EU/μg DNA in the Limulus Amebocyte Lysate assay (QCL 1000, Lonza, USA). Recombinant mouse GM-CSF (Cat #315-03-20) was purchased from Peprotech, Germany. Poly (I:C) (Cat #tlrl-pic), Imiquimod (Cat #tlrl-imq) and CpG ODN 2395 (Cat #tlrl-2395-1) were purchased from Invivogen, USA. The Alexa Fluor 647 dextran (Cat #D22914) was purchased from Molecular Probes, USA.

### Immunization of mouse

C57BL/6 and BALB/c mice were bred and housed at the animal facility, JNCASR. The experiments using animals were performed in strict accordance with the protocols reviewed and approved by the Institutional Animal Ethics Committee, JNCASR (Permit certificate: RUK 003) as per the guidelines of the Committee for the Purpose of Control and Supervision of Experiments on Animals (CPCSEA/201/Go/Re/S/2000), the Government of India. The plasmid DNA was prepared in endotoxin-free cell therapy system grade Dulbecco’s phosphate buffered saline (PBS) (A1285601, ThermoFisher Scientific, USA) and the injections were administered in a volume of 100 μl. For the IM injection, the fur was removed using a sterile scalpel and the DNA was injected into the tibialis anterior muscle. The NF-ID injections were administered in the right flank region just above the tail after clipping the fur using a hair-clipper. The mice were sedated by an intra-peritoneal injection of ketamine-HCl (20 mg/kg) before the immunization. The NF-ID immunization was performed using the B2000 device fitted with a syringe and the adaptor #2 (Bioject Medical Technologies Inc, USA) by administering the entire 100 μl volume at one site following the manufacturer’s guidelines (http://www.bioject.com/products/b2000-info). In the needle-syringe mode, the 100 μl volume was distributed to two sites after shaving the skin. One milliliter syringes with 31G needles were used for the injection.

### Preparation of single cell suspensions for flow cytometry

The spleens and the lymph nodes harvested from immunized mice were dissociated into single cell suspensions with the back of a sterile plunger. The cell suspension was washed once with PBS prior to the lysis of the RBC. The cells collected from a single spleen or a pool of four lymph nodes were incubated for 4 min in 4 ml of ammonium chloride lysis buffer (ACK lysis buffer: 150 mM NH_4_Cl, 10 mM KHCO_3_ and 100 μM EDTA) in 50 ml conical-bottomed tubes to lyse the RBC. The cell suspension was immediately diluted ten-fold using cold RPMI medium (AL157, Himedia, India) supplemented with 2% fetal bovine serum (FBS, Cat #26140079, Gibco URL, Invitrogen, USA) and centrifuged at 1,500 rpm to harvest the leukocytes. The cell suspension was washed extensively using RPMI Supplemented with 2% FBS. The cells were suspended in the complete RPMI medium supplemented with 10% FBS, 2 mM Glutamine, 50 μM β-mercaptoethanol, 100 IU/ml penicillin, and 100 μg/ml Streptomycin. The mice were bled through the retro-orbital plexus and the blood drops were collected into plastic vials containing K2-EDTA solution (0.1% w/v). For some assays, blood from multiple mice was pooled up to 1 ml. The blood samples were incubated at room temperature for 1 h, the plasma was collected from the top of the vials, and the cells were resuspended in 4 ml of the ACK lysis buffer for 4 min to lyse the RBC as described above. The cells were diluted to 20 ml with PBS and centrifuged. The RBC lysis step was repeated once using 3 ml of the ACK lysis buffer. The cells were washed twice using each time 5 ml of RPMI supplemented with 2% of FBS. The cells were finally suspended in complete RPMI at a density of 10 million per ml for flow cytometry.

### Intracellular cytokine staining and the CD107a degranulation assay

The intracellular cytokine staining (ICS) and the CD107a degranulation assays were performed as reported previously^66^. Briefly, one million leukocytes (depleted of RBC) were plated in 96-cluster, V-bottomed wells and stimulated *in vitro* for 6 h with DMSO (0.1% v/v), a peptide pool comprising of 15-mers spanning the HIV-1 consensus subtype C Gag (each peptide at 0.5 μg/ml), or Con A (0.8 μg/ml) in the presence of anti-CD28 (Cat #553295) and anti-CD49d (Cat #553314) (1 μg/ml each) antibodies. For the CD107a degranulation assay, anti-CD107a-PE (Cat #558661) antibody was added to the medium right at the stimulation phase as per the protocol described previously^25^. Two hours following the activation, Brefeldin A (ICS) or Monensin (CD107a degranulation) was added to the cells at a final concentration of 10 μg/ml to block protein secretion. At the end of the incubation, for the ICS assay, the cells were surface stained with anti-CD4-PerCP (Cat #553052) and anti-CD3-APC (Cat #553066). Following fixing and permeabilization using BD Cytofix/Cytoperm (Cat #554722), the intracellular cytokines were stained with anti-TNF-α-FITC (Cat #554419) and anti-IFN-γ-PE (Cat #554412). For the CD107a degranulation assay, the cells were surface stained with anti-CD8-PerCP (Cat #553038) and anti-CD3-APC. The 15-mer peptide pool spanning the consensus subtype C Gag (Cat #8118) was obtained from the NIH-AIDS reagent program. All the antibodies used in the study were obtained from BD Biosciences, USA and titrated prior to the experiments. The stained cells were resuspended in 250 μl of 0.4% paraformaldehyde and transferred to 5 ml round bottomed tubes. To each tube, 150 μl of sheath fluid was added and the samples were acquired using the FACS Aria III or FACS Caliber instruments (BD Biosciences, USA). We acquired and analyzed 0.1–0.2 million events for each assay. The flow cytometry data were analyzed using FlowJo 7.6.1 software for Windows (TreeStar, USA). The live lymphocyte population was determined based on the scattering properties and the T cells were gated on CD3+ staining. The T cells were further delineated into CD8 and CD4 cells by gating on the CD4 negative and positive populations, respectively, in the ICS assay. In the CD107a degranulation assay, the CD8 T cells were identified as the CD3+CD8+ population.

### The lymphocyte proliferation assay

The lymphocyte proliferation assay using the Carboxyfluorescein succinimidyl ester (CFSE) dye was performed as previously reported^67^. The leukocytes were suspended at a density of 20 million cells per ml for the labeling. The CFSE dye (Cat #21888, Sigma) was dissolved at a concentration of 5 mM in DMSO. The cell suspension was centrifuged at 2,000 rpm for 4 min and the cells were resuspended in 1 ml of PBS containing 5% (v/v) of FBS. To the cell suspension in 15 ml conical bottomed tubes, 100 μl of 50 μM CFSE solution (diluted in PBS) was added and the samples were vortexed immediately to facilitate homogenous dissolution of the CFSE solution at a final concentration of 5 μM. The cells were allowed to stand for 5 min at room temperature and diluted with ten volumes of PBS containing 5% of FBS, and centrifuged at 2,000 rpm for 4 min. The cell pellets were washed two times with PBS containing 5% of FBS and resuspended in the complete RPMI medium at a density of 2 million per ml. One hundred microlitre of the cell suspension containing 0.2 million cells were plated in round-bottomed 96-w plates and stimulated with DMSO (0.1% v/v), a peptide pool comprising 15-mers spanning HIV-1 consensus subtype C Gag (each peptide at 0.5 μg/ml), or Con A (0.8 μg/ml) in the presence of anti-CD28 and anti-CD49d (1 μg/ml each) antibodies for 4 d. Unstimulated CFSE-labelled cells were used to define the quadrants for measuring the CFSE dilution. At the end of 4 d of incubation, the cells were surface stained with anti-CD8-PerCP and anti-CD3-APC for the flow cytometry analysis. For the CFSE-dilution analysis, a total of 20,000 events were acquired. The live lymphocyte population was determined based on the scattering properties and the cells were gated for CD3+CD8+ and CD3+CD8- cells to identify the CD8 and CD4 T cells, respectively.

### Enzyme-linked immunosorbent assay (ELISA)

The antigen-specific antibody responses were measured using an indirect ELISA protocol. One hundred nanogram of the antigen (recombinant p24, Tat or firefly luciferase protein) was coated on Maxisorp Nunc-immuno modules (Cat #469949, Thermo Scientific, USA) by incubating 50 μl of the protein solution in PBS overnight at 4 °C. The next morning, the wells were washed once with PBS and blocked with 3% bovine serum albumin (BSA) dissolved in PBS. After blocking for 2 h at room temperature, 100 μl of the serum samples diluted in 1% BSA containing PBST (PBS with 0.2% v/v Tween 20) were added to appropriate wells and incubated for 2 h at 37 °C. The wells were then washed with PBS containing 0.05% v/v Tween 20. The wells were incubated with one hundred microlitres of suitably diluted secondary antibodies, anti-mouse IgG (Cat #82114068001, GeNei, India) or anti-mouse IgM (Cat #626820, Invitrogen, USA) conjugated to horse radish peroxidase (HRP), at room temperature for 1 h. The wells were then washed and incubated with 100 μl of TMB substrate (Cat #50-76-00, KPL Laboratory, USA). The reaction was stopped using 50 μl of 2 N H_2_SO_4_ after 15 min and the absorbance values were measured at 450 nm. The background absorbance values of the wells were measured at 655 nm and subtracted from the assay values.

### The recombinant protein production and purification

The HIV-1 p24 protein coding sequence was amplified using the plasmid pET21b+ - p55 plasmid harboring the *gag* sequence derived from the HIV-1 subtype C molecular clone INDIE-C1 (Genbank accession number: AB023804). The gene sequence of the p24 protein was amplified using the primer pair N2108: 5′-AAGGAGA*CATATG*CCTATAGTGCAGAATCTCCAAGGGC-3′ and N2109: 5′-GATTGC*CTCGAG*AGCCAACACTCTTGCTTTGTGGCCAG-3′ using the conditions 94^30”^-55^45”^-72^1’^ for 15 cycles. The Histidine-tag was added to the p24 cassette at the C-terminal end when the amplified product was cloned directionally into NdeI and XhoI sites of the pET21b+ expression vector (Cat #69741-3, Novagen, USA). The *E. coli* BL21(DE3) strain was transformed with the recombinant p24-expression vector. The protein expression was induced in transformed bacterial cells at a density of 0.45 OD_600nm_ with 1 mM IPTG. The cells were incubated with shaking for 4 h at 37 °C. The bacterial cells harvested following the induction protocol were resuspended in 50 mM phosphate buffer containing 150 mM NaCl (lysis buffer, 20 ml for a litre of culture volume). The cells were lysed by sonication and the cell debris was removed by centrifugation at 16,000 rpm for 30 min. The cell lysate containing the protein was mixed with equilibrated Cobalt chelating resin, 0.5 ml per 20 ml lysate (Cat #786-286, GBiosciences, USA) and gently rocked at 4 °C for 2 h using a rotating shaker. The lysate-bead suspension was then poured into a chromatography column and the flow through was drained. The beads were washed twice with the lysis buffer containing 10 mM imidazole and 2% v/v Triton X-100 followed by a wash with lysis buffer containing only 10 mM imidazole. The bound protein was eluted using PBS containing 150 mM imidazole. The sample was passed through an Amicon filter (10 kDa MWCO, Cat #UFC901008, Merck Millipore, USA) three times to reduce the imidazole concentration. The *tat* expression vector, pET21b-Tat, encoding the viral gene segment derived from a primary Indian clinical sample was reported previously (8). Tat protein was expressed in bacteria and purified as per the reported protocol^68^. The His-tagged Tat protein was purified using a tandem Ni-NTA affinity chromatography followed by a cation-exchange chromatography as reported. The purity of the recombinant proteins was confirmed by SDS-PAGE and the proteins were stored at −80 °C as multiple 100 μl aliquots. The recombinant firefly luciferase protein was purchased from a commercial source (Cat #E170A, Promega Corporation, USA).

### The luciferase assay

The Gaussia and firefly luciferase activities were measured from the sera and the tissues extracts, respectively. For the GLuc assay, the serum samples were diluted 10-fold in PBS and 10 μl of the diluted samples was mixed with 10 μl of the commercial substrate solution (Cat #E3300L, BioLux Gaussia luciferase assay kit, NEB, USA). The values of light intensity were measured after a delay time of 15 s with an integration time of 10 s using the Varioskan Flash multimode reader (Thermo Scientific, USA). The skin or the muscle tissues harvested at different time points were snap frozen in liquid nitrogen and stored at −80 °C until further processing. The frozen tissues were pulverized into fine powder using a sterile pestle and mortar under liquid nitrogen. The samples were resuspended in 1X-passive lysis buffer (Cat #E1941, Bright-Glo luciferase assay system, Promega Corporation, USA), vortexed for 15 min at 4 °C and subjected to three cycles of freezing at −192 °C and thawing at 37 °C. The tissue extracts were clarified by centrifugation at 4 °C for 30 min. The protein concentration of the samples was measured using the BCA assay (Cat #23227, Thermo Scientific, USA) and the concentration was adjusted to 5 mg/ml with 1X-passive lysis buffer. Using the Spectramax L microplate luminometer (Molecular devices, USA), the luciferase activity was measured in 10 μl of the protein lysates after the addition of 10 μl D-luciferin substrate (Cat #E2610, Bright-Glo^TM^ luciferase assay system, Promega Corporation, USA). Delay and integration times are 0.2 and 10 s, respectively. The luciferase assays were carried out in 96-w round-bottomed opaque white plates (Cat #CLS3789-100EA, Sigma-Aldrich, USA).

### The quantitative real-time PCR analysis

The total DNA from the skin and muscle tissues was isolated following the protocol reported previously^69^. In brief, the tissues were pulverized into fine powder in the presence of liquid nitrogen. The tissue powder, up to 100 mg, was dissolved in 500 μl of Tris-Cl buffer (pH 8.0) containing 200 μg/ml of proteinase K and 0.5% w/v SDS and incubated overnight at 65 °C. Following this, 75 μl of 8 M potassium acetate solution was added to each vial and the samples were incubated at 4 °C for 15 min. The samples were extracted with 750 μl of chloroform and the DNA present in the aqueous layer was precipitated with an equal volume of ethanol. The precipitate was washed twice with 70% ethanol and the DNA was dissolved in TE buffer by incubating the samples at 4 °C overnight. A real-time PCR using the SYBR green chemistry (Cat #BIO-98002, SensiFAST^TM^ SYBR No-ROX kit, BIOLINE, Singapore) was performed using 25 ng of the extracted DNA. A 106 bp CMV promoter region was amplified using the primer pair, N2410 CMV forward –5′-CATAGTAACGCCAATAGGGACTTTCC-3′ and N2410 CMV reverse –5′-GGGCGTACTTGGCATATGATACAC-3′. A 120 bp region of the GAPDH gene was amplified using the primers, N1040 GAPDH forward –5′-GAGCTGAACGGGAAGCTCACT-3′ and N1041 GAPDH reverse –5′-CACGTCAGATCCACGACGGACACATTG-3′. The GAPDH amplification served as the normalization control. The standard curves were generated using a range of pCMV-GFP and pGAPDH plasmids dissolved in a buffer containing 50 ng of salmon sperm DNA. The copy number of the CMV or GAPDH region in the template DNA was determined by scatchard analysis using the standard curves. The sensitivity of the CMV and GAPDH PCRs was 100 copies of the respective plasmid DNA. The real-time PCR analyses were performed using the Bio-rad CFX96 real time PCR machine.

### Immunofluorescense analysis of the tissue sections

The dissected skin tissues were embedded in tissue embedding medium (Cat #03801690, Leica Biosystems, Germany) and maintained on dry ice until sectioning. The frozen tissues were sectioned into 10 μm slices using a cryostat (Leica CM-3050-S, Leica Biosystems, Germany) and fixed with 4% paraformaldehyde for 5 min. The fixed tissue sections were washed thrice with 1X-PBS and mounted with Prolong Gold antifade mountant containing DAPI (Cat #P36935, Life technologies, USA). The sections were scanned using the Zeiss LSM 510 laser scanning fluorescence confocal microscope and multiple Z-sections were captured at a magnification of 20X or 63X with the dry or oil immersion objective of 0.5 NA. The final images presented were obtained after the stacking of the Z-sections. The images were examined using the FIJI software.

### Statistics

All the statistical analyses were carried out using GraphPad Prism Version 6.0 for Mac OS X, GraphPad Software, La Jolla California, USA.

## Additional Information

**How to cite this article**: Arunachalam, P. S. *et al.* Toll-Like Receptor 9 Activation Rescues Impaired Antibody Response in Needle-free Intradermal DNA Vaccination. *Sci. Rep.* **6**, 33564; doi: 10.1038/srep33564 (2016).

## Supplementary Material

Supplementary Information
