# Supplementary material for: Toll-Like Receptor 9 Activation Rescues Impaired Antibody Response in Needle-free Intradermal DNA Vaccination
Source: Sci Rep. 2016 Sep 23;6:33564. doi: 10.1038/srep33564 (PMC5034244; doi:10.1038/srep33564)
Supplement: Supplementary Information [file srep33564-s1.pdf]

Toll-Like Receptor 9 Activation Rescues Impaired Antibody Response in Needle-free Intradermal DNA Vaccination

Prabhu S Arunachalam, Ria Mishra, Deepak Selvam, Sravan K Payeli, Richard R Stout and Udaykumar Ranga

Figure S1

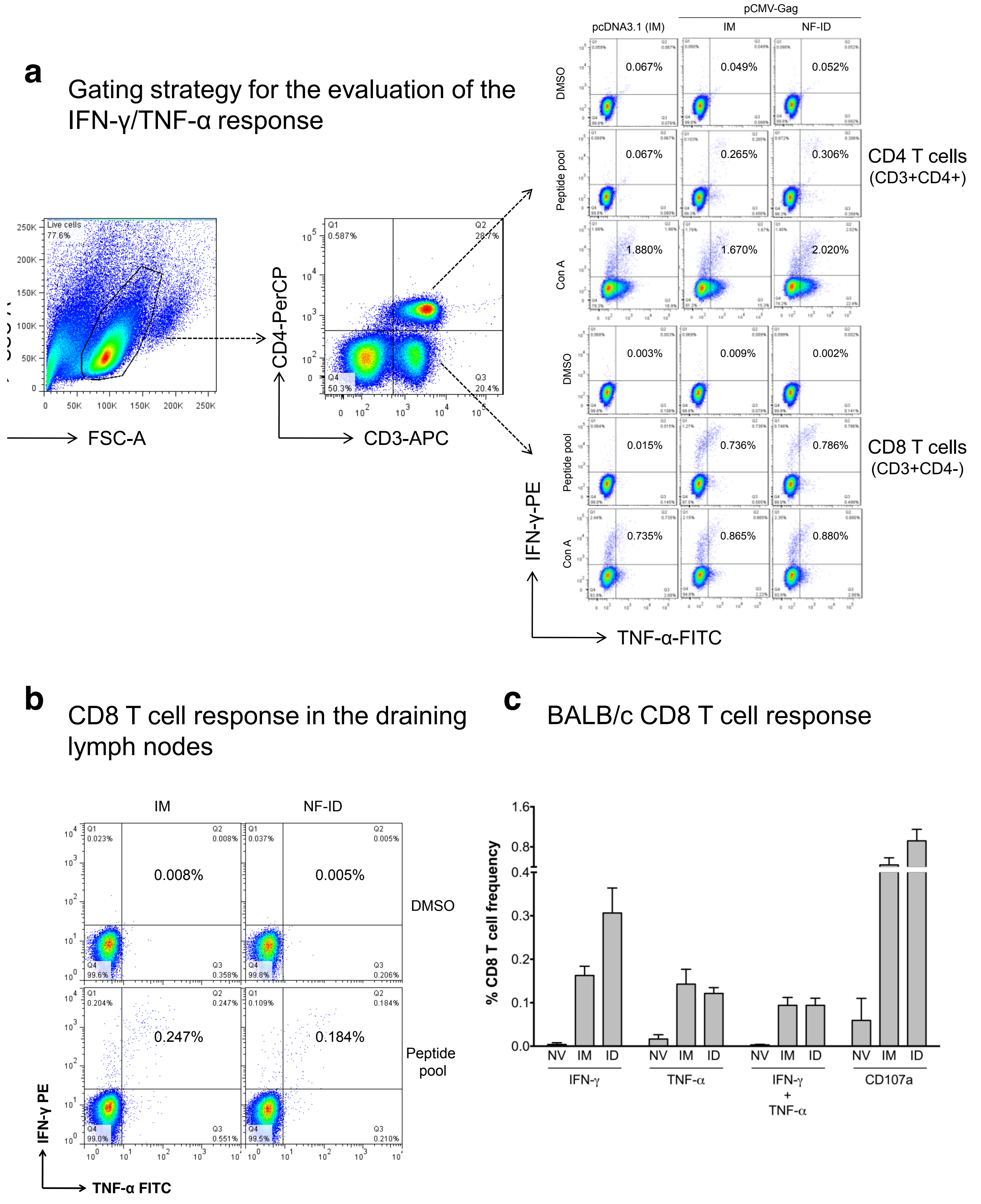

Figure S1 Evaluation of the p24-specific T cell response in the immunized mice. (**a**) Representative plots showing the gating strategy for the flow cytometry analysis of CD3+CD4+ (CD4) and CD3+CD4- (CD8) T cells expressing IFN-γ and TNF-α. (**b**) The flow cytometry analysis of CD3+CD8+ cells from the draining lymph nodes of mice immunized with 50 µg pCMV-Gag vector. The cells derived from the ipsilateral popliteal and inguinal lymph nodes were pooled and analyzed. (**c**) The CD8 T cell responses measured from the BALB/c mice (n=6) as depicted in figure 1. The data are representative of two independent experiments. Unpaired *t* test was used for the statistical comparison.

Figure S2

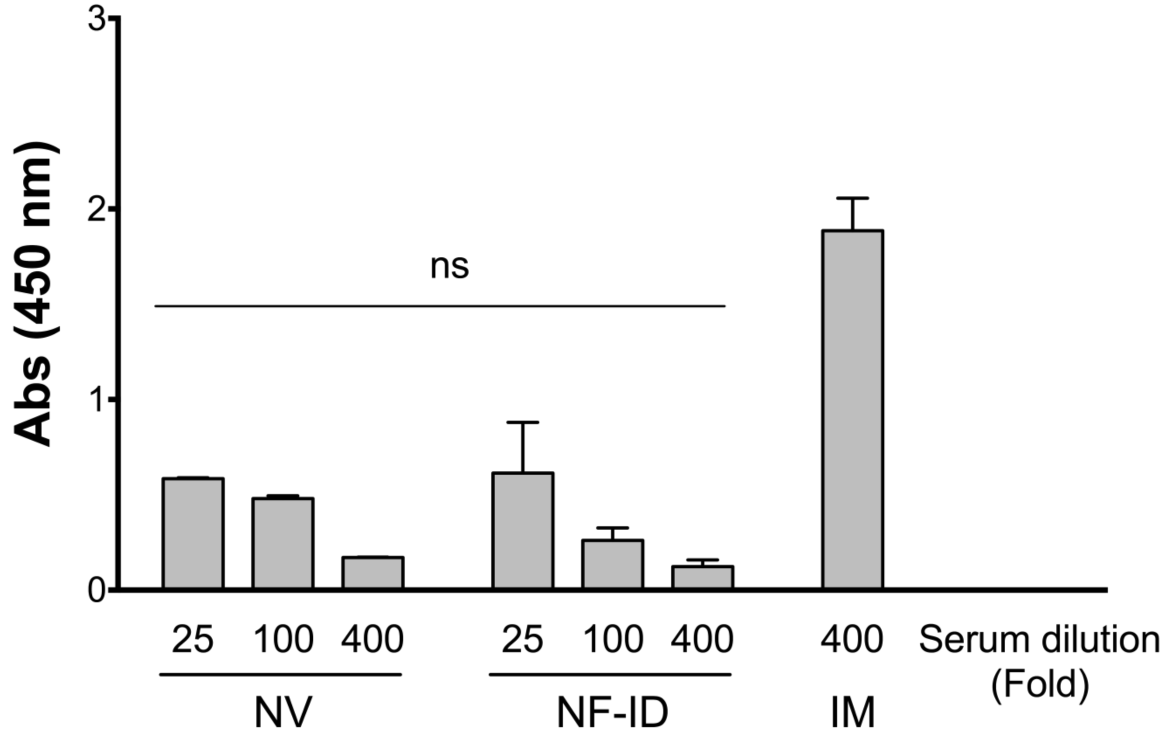

Figure S2 The anti-p24 antibody response in C57BL/6 mice. The sera of the C57BL/6 mice (n=12) immunized as in figure 1A were collected on day 11 following the booster immunization, and the p24-specific antibody response was measured by ELISA. The mean ± SEM absorbance values of the serum samples diluted serially (25 to 100-fold) are plotted.

Figure S3

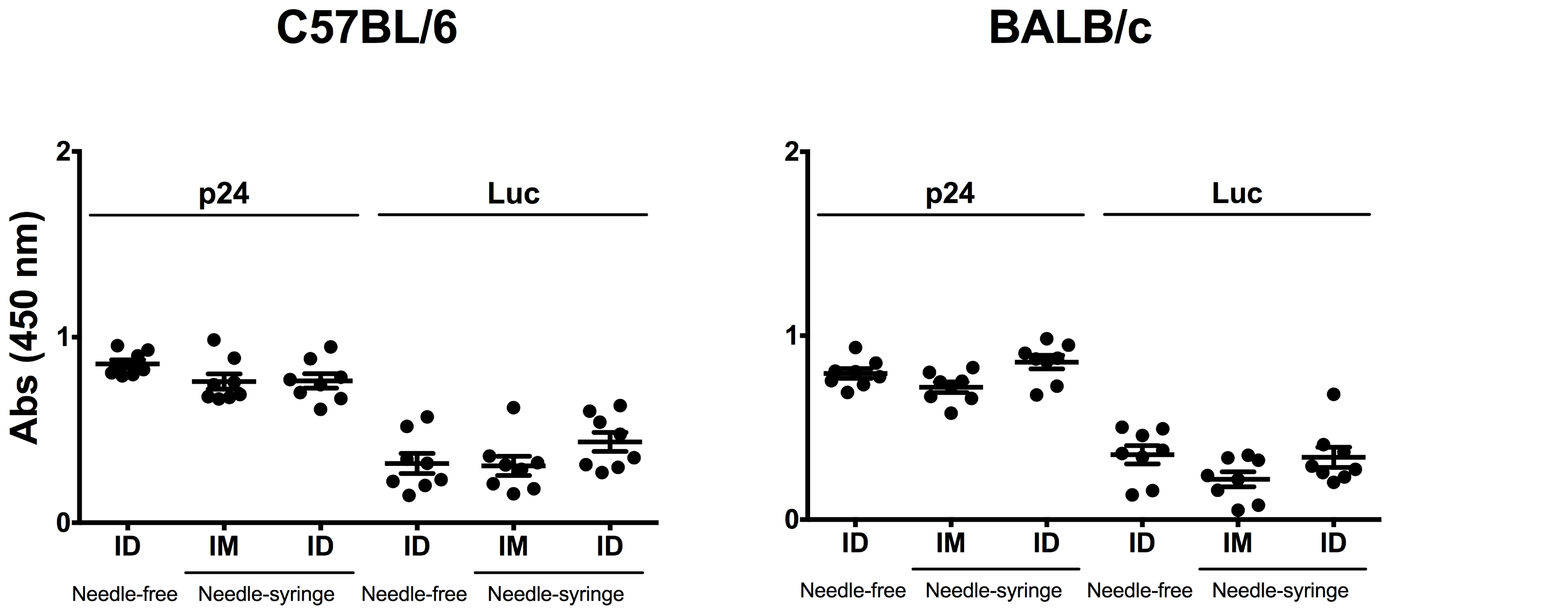

Figure S3 Antigen specificity of the IgM response. The sera of C57BL/6 or BALB/c mice immunized with 50 µg pCMV-Gag expression vector through one of the routes depicted in the X-axis were diluted 100-fold and analyzed by ELISA using wells coated with 100 ng of p24, the specific antigen or firefly luciferase, a non-specific antigen. The mean absorbance ± SEM are plotted. Each dot represents the serum sample of a mouse.

Figure S4

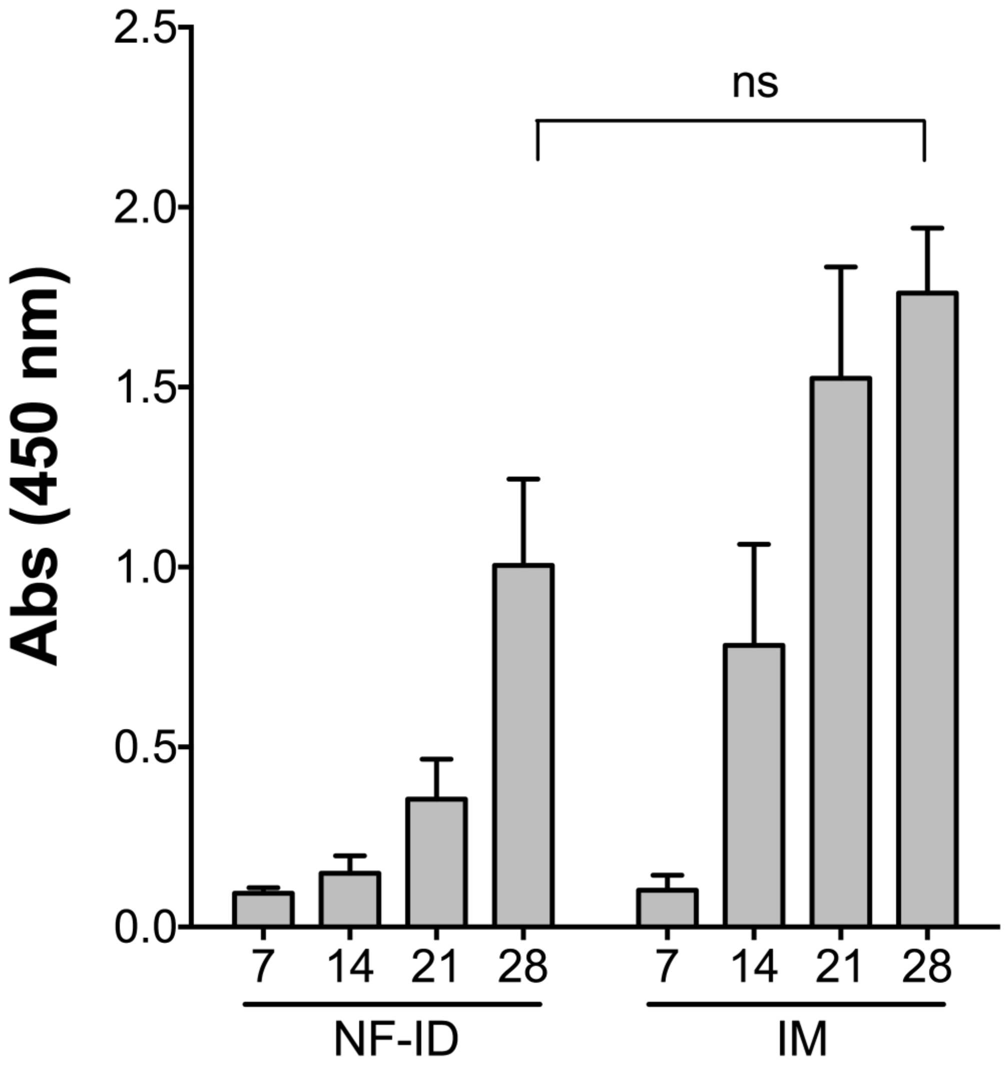

Figure S4 Anti-p24 antibody titers in mice administered multiple booster immunizations of 15 µg of the pCMV-Gag expression vector. BALB/c mice (n=4) per group were immunized with 15 µg pCMV-Gag expression vector using the one-prime-three-booster or one-prime-three booster regimen as depicted in figure 5. Means ± SEM of the raw absorbance values obtained from the ELISA are plotted.

Figure S5

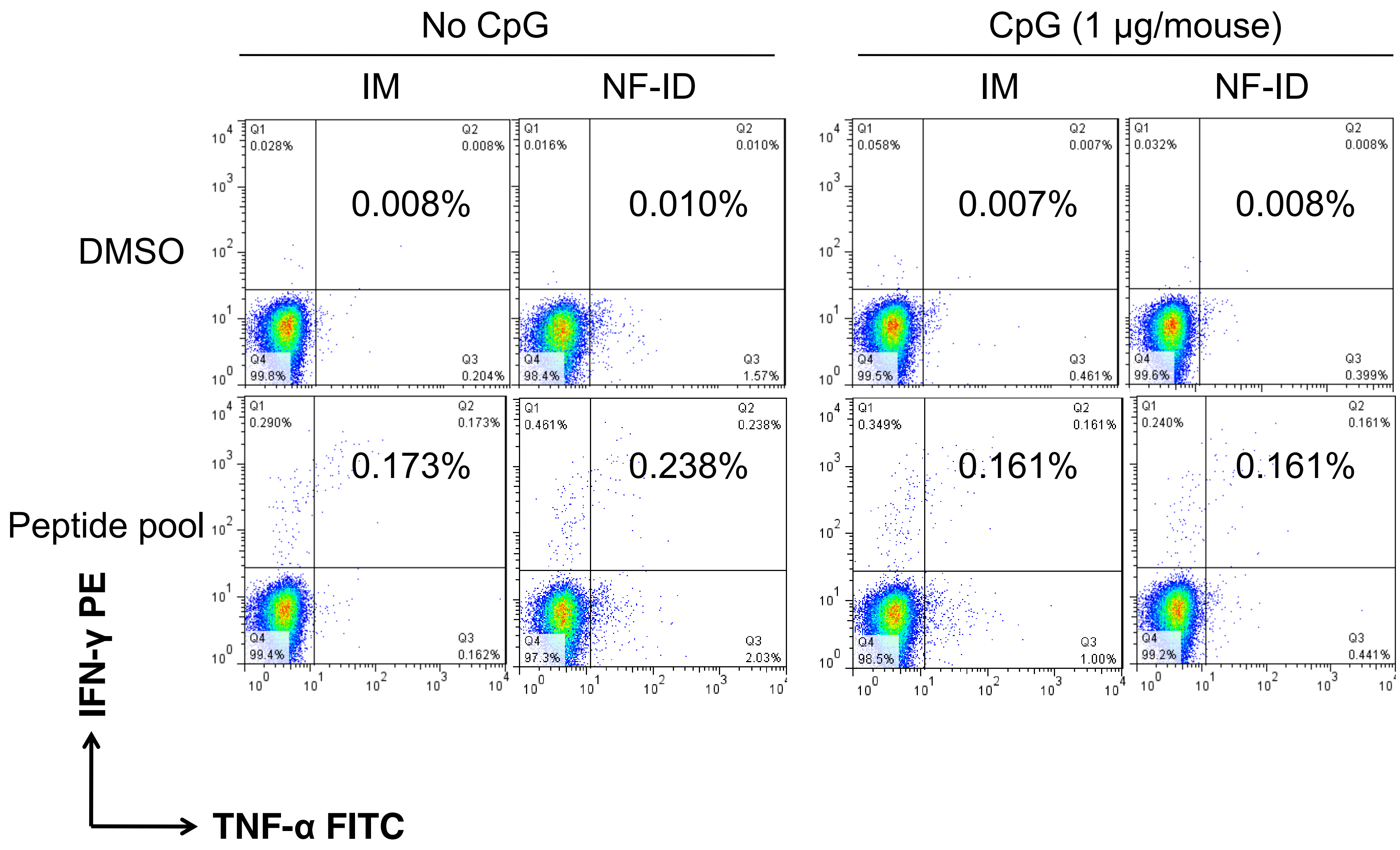

Figure S5 The T cell responses measured from the peripheral blood leukocytes of mice co-immunized with the pCMV Gag vector and CpG ODN 2395. C57BL/6 mice immunized with 50 µg pCMV-Gag vector with or without 1 µg CpG ODN were bled on day 10 following the booster immunization and analyzed for IFN-γ/TNF-α double-positive T cell response using the ICS technique. The flow cytometry profiles of cells gated on the CD3+CD8+ subset are presented.
